# Cytokines and Chemokines in Cerebral Malaria Pathogenesis

**DOI:** 10.3389/fcimb.2017.00324

**Published:** 2017-07-20

**Authors:** Josefine Dunst, Faustin Kamena, Kai Matuschewski

**Affiliations:** ^1^Parasitology Unit, Max Planck Institute for Infection Biology Berlin, Germany; ^2^Institute of Chemistry and Biochemistry, Free University Berlin, Germany; ^3^Molecular Parasitology, Institute of Biology, Humboldt University Berlin, Germany

**Keywords:** malaria, *Plasmodium*, cerebral malaria, cytokines, chemokines, endothelial activation, blood-brain barrier, neuroinflammation

## Abstract

Cerebral malaria is among the major causes of malaria-associated mortality and effective adjunctive therapeutic strategies are currently lacking. Central pathophysiological processes involved in the development of cerebral malaria include an imbalance of pro- and anti-inflammatory responses to *Plasmodium* infection, endothelial cell activation, and loss of blood-brain barrier integrity. However, the sequence of events, which initiates these pathophysiological processes as well as the contribution of their complex interplay to the development of cerebral malaria remain incompletely understood. Several cytokines and chemokines have repeatedly been associated with cerebral malaria severity. Increased levels of these inflammatory mediators could account for the sequestration of leukocytes in the cerebral microvasculature present during cerebral malaria, thereby contributing to an amplification of local inflammation and promoting cerebral malaria pathogenesis. Herein, we highlight the current knowledge on the contribution of cytokines and chemokines to the pathogenesis of cerebral malaria with particular emphasis on their roles in endothelial activation and leukocyte recruitment, as well as their implication in the progression to blood-brain barrier permeability and neuroinflammation, in both human cerebral malaria and in the murine experimental cerebral malaria model. A better molecular understanding of these processes could provide the basis for evidence-based development of adjunct therapies and the definition of diagnostic markers of disease progression.

## Introduction

Malaria is one of the most prevalent infectious diseases worldwide and contributes considerably to the global disease burden. With ~200 million new cases and an estimated 430,000 deaths annually (WHO, 2016), malaria remains the most important vector-borne infectious disease. The human-adapted *Plasmodium* species *P. falciparum* and *P. vivax* account for the majority of malaria cases and are transmitted by the bite of an infective *Anopheles* mosquito. Despite considerable progress in malaria eradication over the past 15 years (WHO, 2016), efforts are hampered by emerging *Plasmodium* resistance to commonly used anti-malaria drugs (Cui et al., 2015) and limited efficacy of the currently most advanced vaccine candidate (White et al., 2015; Olotu et al., 2016).

Among infections by human host-adapted *Plasmodium* species, *P. falciparum* infections are most likely to progress to organ-related pathology and severe malaria, and thereby account for the vast majority of malaria-associated fatalities. Along with severe anemia and respiratory distress, cerebral malaria (CM) is one of the major manifestations of severe malaria (Haldar et al., 2007). CM manifests with impaired consciousness and coma in both children and adults (Idro et al., 2010), while other clinical features differ. In addition to the characteristic diffuse encephalopathy, retinal abnormalities are frequent in children and less common in adults with CM (Beare et al., 2006; Idro et al., 2010). In contrast, CM in adults is accompanied by multi-organ disorder including renal failure and pulmonary edema, which are less frequently observed in children suffering from CM (Idro et al., 2010). Although anti-malaria treatment using artesunate was reported to improve CM outcome in children and adults (Dondorp et al., 2005, 2010), the case-fatality rate of pediatric CM is approximately 20% (Haldar et al., 2007) and sustained cognitive and/or neurological impairment may occur (John et al., 2008a). Consequently, treatment strategies, which not only target the parasite but also other mechanisms underlying CM pathogenesis, need to be developed. Since the pathogenesis of CM is still incompletely understood, further investigations are an important medical research priority, especially in the context of adjunctive therapies. Since accumulating evidence indicates that an imbalance in pro- and anti-inflammatory immune responses partially contributes to CM pathogenesis, such therapeutic approaches could target cytokines and chemokines associated with CM severity. Cytokines are polypeptides, which mediate and generate inflammatory responses. Along with their contribution to disease pathogenesis in general, cytokines also exert physiological roles at lower concentrations (Clark and Vissel, 2017). Chemokines are chemotactic cytokines, which recruit lymphocytes and monocytes to the site of pathogen encounter by binding to their respective chemokine receptor (Griffith et al., 2014). Given that leukocytes were found to sequester in the microvasculature of the brain in human CM and murine ECM (Hunt and Grau, 2003), local chemokine gradients may mediate leukocyte recruitment and thus promote CM pathogenesis. Chemokines exert their function through binding to their respective G protein-coupled chemokine receptors (Table 1), which induces activation of phosphatidylinositol 3-kinase (PI3K) and Rho GTPase signaling pathways, thus leading to F-actin polymerization and migration (Viola and Luster, 2008).

**Table 1:** Selected human and murine chemokines and their receptors^a^.

**Chemokine**	**Other names**	**Gene**		**Receptors**	**Key function**
		**Human**	**Mouse**		
CCL2	MCP-1 JE (mouse)	*CCL2*	*Ccl2*	CCR2	Inflammatory monocyte trafficking
CCL3	MIP-1α	*CCL3*	*Ccl3*	CCR1, CCR5	Macrophage and NK cell migration, T cell-DC interactions
CCL4	MIP-1β	*CCL4*	*Ccl4*	CCR5	
CCL5	RANTES	*CCL5*	*Ccl5*	CCR1, CCR3, CCR5	
CCL11	Eotaxin	*CCL11*	*Ccl11*	CCR3, CCR5	Eosinophil and basophil migration
CCL20	MIP-3α	*CCL20*	*Ccl20*	CCR6	Th17 responses, B cell and DC homing
CXCL1	GROα Gm1960 (mouse)	*CXCL1*	*Cxcl3*	CXCR2	Neutrophil trafficking
CXCL3	GROγ KC (mouse)	*CXCL3*	*Cxcl1*	CXCR2	
CXCL4	PF4	*PF4*	–	CXCR3-B	Procoagulant
CXCL4L1	PF4V1	*PF4V1*	*Pf4*	CXCR3-B	
CXCL8	IL-8	*IL-8*	–	CXCR1, CXCR2	Neutrophil trafficking
CXCL9	MIG	*CXCL9*	*Cxcl9*	CXCR3	T cell and NK cell trafficking
CXCL10	IP-10	*CXCL10*	*Cxcl10*	CXCR3	

a *Modified from Zlotnik and Yoshie (2012) and Griffith et al. (2014)*.

**Figure 1:**
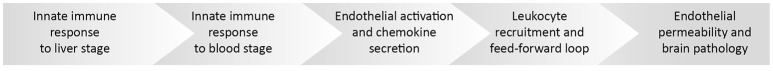
Overview of a potential inflammatory cascade culminating in cerebral malaria pathology. Five consecutive events shape the outcome of a *Plasmodium* infection and contribute to cerebral malaria. During *Plasmodium* infection of the mammalian host, two consecutive parasite replication phases in the liver and red blood cells lead to distinct innate responses, which modulate downstream parasite/host cell interactions. Upon parasite accumulation in the microvasculature, endothelial cells become activated, leading to enhanced chemokine secretion, which in turn enhances leukocyte recruitment. Acute pathology is caused by permeabilization of the endothelial barrier. See Figures 2–6 for the central roles of chemokines and cytokines in the individual events.

In this review, we highlight findings from both experimental murine models and natural human infections, and assess the current knowledge on the role of host cytokine and chemokine responses in the severe malaria complication of cerebral malaria. We also emphasize the potential inflammatory cascade resulting from *Plasmodium* life cycle progression after sporozoite inoculation and ultimately culminating in cerebral malaria pathology (Figure 1).

## Current concepts in cerebral malaria pathogenesis

Two central concepts to explain CM pathogenesis have evolved and they are likely mutually dependent- the vascular occlusion hypothesis and the inflammation hypothesis (Storm and Craig, 2014).

The concept of vascular occlusion leading to CM is based on the ability of mature *P. falciparum*-infected erythrocytes to sequester in the microvasculature through binding of *P. falciparum* erythrocyte membrane protein 1 (*Pf* EMP1) present on the erythrocyte surface to endothelial cell surface proteins, such as intercellular adhesion molecule 1 (ICAM-1), vascular cell adhesion molecule 1 (VCAM-1), cluster of differentiation 36 (CD36), or endothelial protein C receptor (EPCR) (Pasloske and Howard, 1994; Chen et al., 2000; Rowe et al., 2009; Smith et al., 2013; Turner et al., 2013; Lennartz et al., 2017). Sequestration occurs in various organs and, along with increased rigidity of erythrocytes, is believed to cause vascular occlusion (Dondorp et al., 2004). Additionally, microvascular obstruction during *P. falciparum* infection may be worsened by the formation of rosettes and clumps (Chen et al., 2000; Rowe et al., 2009; Adams et al., 2014), i.e., the binding of uninfected erythrocytes by infected erythrocytes (Handunnetti et al., 1989), and aggregation of infected erythrocytes and platelets (Pain et al., 2001), respectively. These events may cause a reduction in microvascular blood flow, ischemia, and tissue hypoxia (Medana and Turner, 2006), thereby accounting for cerebral pathology. Reduced vessel perfusion and occlusion was indeed observed by fluorescein angiography of the retina in pediatric CM cases (Beare et al., 2009) and by *in vivo* imaging of the microcirculation in adult patients with CM or other manifestations of severe malaria (Dondorp et al., 2008), although further studies are needed to determine the underlying cause of these observations. Additionally, sequestration of *P. falciparum*-infected erythrocytes was observed in the cerebral microvasculature of CM patients in *post mortem* brain histology studies (Ponsford et al., 2012; Milner et al., 2014, 2015).

Despite accumulating evidence, the relevance of sequestration in the development of CM is still incompletely understood, since the degree of sequestration in brains of non-fatal CM cases cannot be investigated non-invasively and is, thus, unspecified (Miller et al., 2002). Occasionally, fatal CM cases present little sequestration and vessel occlusion similar to non-CM severe malaria cases (Ponsford et al., 2012). Moreover, isolated CM cases were reported in children and adults with confirmed *P. vivax* mono-infections in India (Kochar et al., 2009), although *P. vivax* is unlikely to sequester in the microvasculature since late-stage *P. vivax*-infected erythrocytes are present in peripheral blood (Anstey et al., 2009). Together, cerebral malaria occasionally develops in a few *P. vivax* infections without obvious signs of sequestration *in vivo* or microvascular obstruction.

Given the unresolved role of sequestration in the pathogenesis of CM, additional factors may determine disease severity. Notably, other infectious diseases that result in systemic inflammation and fever also progress to severe forms, including neurological complications such as sepsis-associated encephalopathy (De Backer et al., 2002; Clark et al., 2004). Strikingly, systemic cytokine levels have been described to correlate with disease severity in malaria as well as sepsis (Prakash et al., 2006; Bozza et al., 2007). These findings corroborate an earlier proposal that an imbalance in pro- and anti-inflammatory immune responses triggers immune-induced pathology and might be a leading cause of CM pathogenesis, which may be further amplified by sequestration (Clark and Rockett, 1994). In addition to inflammation and sequestration, CM is associated with endothelial activation and increased blood-brain barrier permeability, and these processes might act reciprocally and have synergistic effects (van der Heyde et al., 2006). In line with this notion, certain *Pf* EMP1 variants, which are associated with CM, were described to compete with activated protein C in binding to EPCR (Turner et al., 2013; Bernabeu et al., 2016). Therefore, the anti-coagulant, anti-inflammatory, cytoprotective properties, which are induced upon interaction of protein C with EPCR, might be impeded by *Pf* EMP1-EPCR interaction and consequently, disease mechanisms may be further exacerbated (Bernabeu and Smith, 2017; Wassmer and Grau, 2017). However, the impact of the interaction between *Pf* EMP1 and EPCR on inflammation and coagulation remains to be demonstrated.

Insights into the mechanisms underlying CM in humans are limited and mostly based on *post mortem* histopathology or correlations of serum parameters with disease outcome (Hunt and Grau, 2003). Despite potential differences in human and murine CM pathogenesis (Riley et al., 2010; Craig et al., 2012), *P. berghei* (strain ANKA) infection reliably causes signature symptoms of CM in susceptible C57BL/6 mice (de Souza and Riley, 2002; Hunt and Grau, 2003). This host-parasite combination is a widely used and well-established murine model for CM, termed experimental cerebral malaria (ECM), which permits mechanistic studies (Craig et al., 2012). Consequently, studies highlighted in this review include reports on human cerebral malaria cases combined with mechanistic insights based on the murine ECM model and *in vitro* studies.

## Innate immune activation during liver stage development

**Figure 2:**
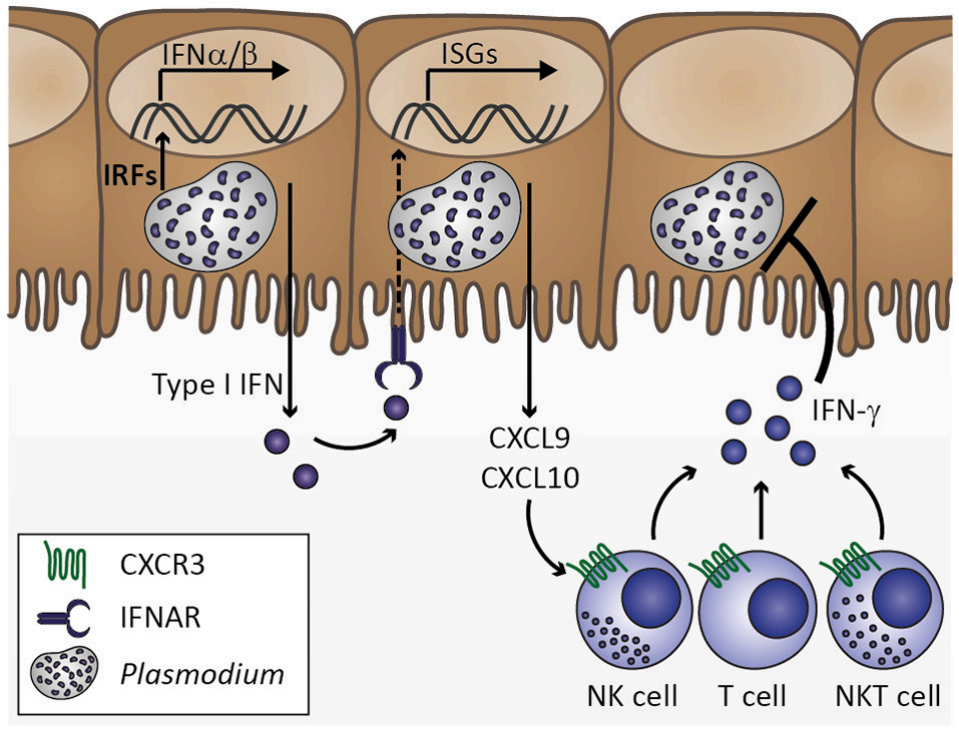
Innate immune response to *Plasmodium* liver stage infection. *Plasmodium* infection of hepatocytes activates interferon regulatory factors (IRF), which induce transcription of type I interferons (IFN) IFNα and IFNβ. Secretion of type I IFNs activates IFNα/β receptor IFNAR in an autocrine or paracrine manner. IFNAR signaling results in transcription of IFN-stimulated genes (ISGs), which includes chemokines, such as CXCL9 and CXCL10. Upon secretion from hepatocytes, these chemokines might recruit cells expressing the corresponding chemokine receptor CXCR3, including natural killer (NK), T, and NKT cells. Upon activation by type I IFN at the site of infection, these cell types could contribute to limiting *Plasmodium* liver stage expansion by IFN-γ secretion. Based on Liehl et al. (2014) and Miller et al. (2014).

Upon transmission by the bite of an infective *Anopheles* mosquito, *Plasmodium* sporozoites rapidly migrate to the liver, invade hepatocytes and develop into thousands of merozoites. Since this developmental stage of *Plasmodium* parasites is clinically silent, the immune response mounted by the host in order to limit parasite expansion during liver stage development remains largely unexplored (Hafalla et al., 2011). However, it seems likely that sporozoite and liver stage recognition primes the innate immune system locally and well below the pyrogenic threshold. In fact, innate immune cells have occasionally been observed to surround *P. berghei*-infected hepatocytes, indicating that *Plasmodium* does not remain undetected during liver stage development (Liehl and Mota, 2012). Additionally, a type I interferon (IFN) response is induced in livers of mice infected with *P. yoelii* or *P. berghei* (Liehl et al., 2014; Miller et al., 2014). Such an initial type I IFN response might induce chemokines, including IFN-γ-inducible protein 10 (IP-10)/CXCL10, which in turn could recruit cells expressing the corresponding chemokine receptor CXCR3, such as T, natural killer (NK), and NKT cells, to the site of infection, which might contribute to the local immune response by IFN-γ secretion (Figure 2; Liehl et al., 2014; Miller et al., 2014). In good agreement, an increase in IFN-γ plasma concentration prior to onset of detectable blood-stage infection was reported in controlled human *P. falciparum* infection (Hermsen et al., 2003). Clearly, the initial cytokine response fails to arrest liver stage development and, thus, does not curtail the proceeding to erythrocyte infection. Interestingly, *P. berghei* sporozoite and blood stage infections result in ECM symptoms in a similar time frame (Kordes et al., 2011), suggesting that immune responses against liver stages might not modulate CM pathogenesis. Whether this first immune response reduces the initial parasite number released into the blood stream and thereby influences the magnitude of early blood stage-induced immune responses remains to be tested.

## Blood stage-induced innate immune responses

Concomitant with the release of merozoites into the bloodstream and infection of erythrocytes, a *Plasmodium* infection progresses from the clinically silent liver stage to the symptomatic blood stage. Merozoites are only very briefly (~60 s) exposed to the immune system before they rapidly enter new erythrocytes (Gilson and Crabb, 2009; Beeson et al., 2016), and blood stage infection is the exclusive cause of malaria symptoms, which is associated with systemic inflammation and fever. Fever is a common and effective host defense against microbial pathogens and swiftly initiated upon the first host-pathogen interaction. The febrile response is likely triggered through a universal mechanism, in which pyrogens, such as the pro-inflammatory cytokines interleukin 1α (IL-1α), IL-1β, IL-6, or tumor necrosis factor (TNF), are secreted by innate immune cells upon recognition of pathogen-associated molecular patterns (PAMPs) or host-derived danger-associated molecular patterns (DAMPs) by pattern recognition receptors (PRRs) (Evans et al., 2015). In *Plasmodium* infection, the characteristic recurrent fever coincides with synchronized rupture of infected erythrocytes in the schizont stage (Oakley et al., 2011). Release of parasite- and host-derived molecules due to erythrocyte rupture was described to induce TNF *in vitro* (Kwiatkowski et al., 1989; Bate and Kwiatkowski, 1994), and peaks in TNF serum concentration were found to coincide with elevated body temperature during *P. vivax* infection (Karunaweera et al., 1992), indicating that malaria fever is elicited by repeated release of PAMPs and DAMPs. Although an increase in core body temperature is associated with resolution of infection (Oakley et al., 2011), such a pro-inflammatory immune response needs to be counterbalanced by anti-inflammatory mechanisms in order to avoid a dysregulated immune response, which might lead to complications such as cerebral malaria.

**Figure 3:**
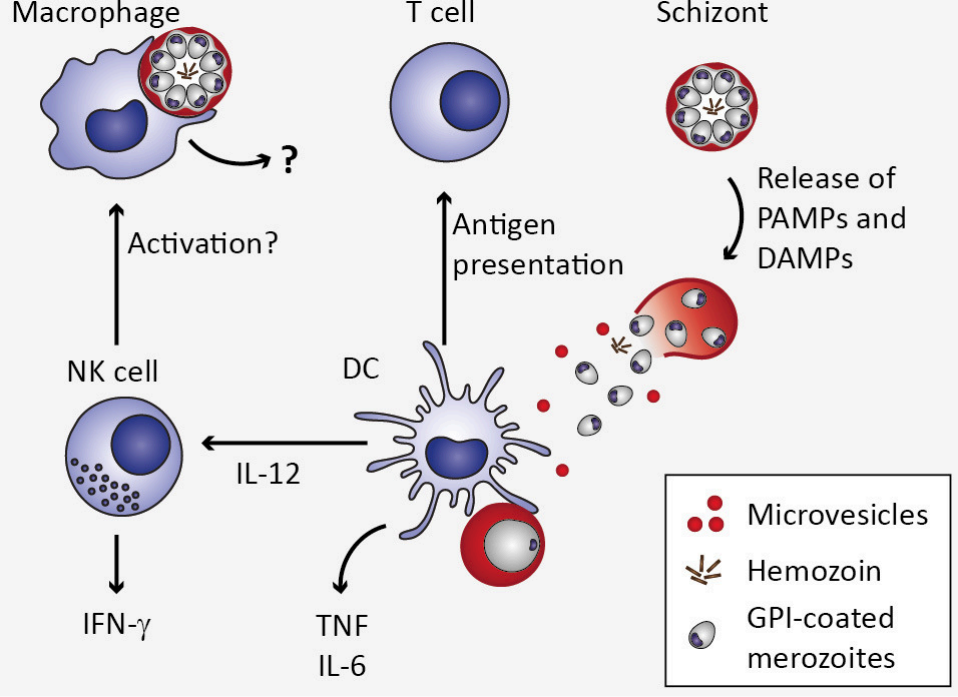
Innate immune response to *Plasmodium* blood stage infection in the spleen. Macrophages as well as dendritic cells (DC) remove infected erythrocytes from the circulation by phagocytosis. In macrophages, uptake of infected erythrocytes might not lead to secretion of pro-inflammatory cytokines due to phagosomal acidification (Wu et al., 2015). Upon rupture of infected erythrocytes, pathogen-associated molecular patterns (PAMPs) and danger-associated molecular patterns (DAMPs) are released, including microvesicles, hemozoin, and glycosylphosphatidylinositols (GPI). These potential PAMPs and DAMPs might be recognized by DC through pattern recognition receptors, resulting in the secretion of interleukin 12 (IL-12), tumor necrosis factor (TNF), and IL-6 (Wu et al., 2015). DC-derived IL-12 might activate natural killer (NK) cells, which in turn secrete interferon γ (IFN-γ) and could thereby activate macrophages (Stevenson and Riley, 2004).

The innate immune system represents the first line of defense against pathogens and mediates recognition and clearance of *Plasmodium* parasites (Figure 3). Cells of the innate immune system such as macrophages and dendritic cells (DCs) as well as non-professional immune cells such as endothelial cells and fibroblasts express PRR. These include Toll-like receptors (TLR), C-type lectin receptors (CLR), Retinoic acid-inducible gene (RIG)-I-like receptors, and NOD-like receptors (NLR), which recognize PAMPs and host-derived DAMPs (Takeuchi and Akira, 2010). Most malaria PAMPs and DAMPs known so far are apparently recognized by TLR. Activation of TLR initiates a signaling cascade including the adaptor protein MyD88 and the transcription factors NF-κB, AP-1, and interferon regulatory factor (IRF). As a consequence, expression of genes encoding pro-inflammatory cytokines such as type I IFN, IFN-γ, IL-6, IL-12, and TNF, is induced (Eriksson et al., 2013; Gazzinelli et al., 2014).

Several *Plasmodium*-derived molecules have been recognized as malaria PAMPs based on their ability to induce cytokine responses *in vitro* (Figure 3). One of the candidate malaria PAMPs are glycosylphosphatidylinositols (GPI). Although GPI are present in all eukaryotic cells where they serve as membrane anchors for certain cell surface proteins, *Plasmodium* GPI are structurally distinct from human GPI (Gowda, 2007). Consequently, *Plasmodium* GPI moieties may be recognized by the host immune system. Indeed, *P. falciparum* GPI were reported to induce the pro-inflammatory cytokines TNF and IL-1β in murine macrophages *in vitro* (Schofield and Hackett, 1993; Tachado et al., 1996). Moreover, purified GPI immobilized on gold particles elicited pronounced TNF responses from murine macrophages *in vitro* (Krishnegowda et al., 2005; Zhu et al., 2009, 2011), which was attributed to recognition of *P. falciparum* GPI through TLR2 or heterodimers of TLR2/1 and TLR2/6 (Krishnegowda et al., 2005; Zhu et al., 2011). However, given that TLR-deficiency did not impair immune responses elicited by *P. berghei* ANKA *in vivo* and did not protect mice from ECM (Togbe et al., 2007; Lepenies et al., 2008), the mechanism of GPI-induced innate immune activation *in vivo* remains to be determined.

Another potential malaria PAMP is hemozoin, an insoluble polymer formed inside the digestive vacuole to detoxify heme and its conjugated redox-active iron, which is released during hemoglobin proteolysis (Francis et al., 1997; Sigala and Goldberg, 2014). Hemozoin becomes accessible during erythrocyte rupture and upon phagocytosis of infected erythrocytes, and has been described to induce expression of pro-inflammatory cytokines, such as TNF and IL-1β, as well as chemokines from human monocytes, murine macrophages (Olivier et al., 2014), and human monocyte-derived DCs (Bujila et al., 2016). Hemozoin or hemozoin-bound nucleic acids are recognized by endosomal TLR9 (Coban et al., 2005; Parroche et al., 2007), cytoplasmic inflammasomes, or cytoplasmic sensors (Kalantari et al., 2014). However, it remains unresolved whether hemozoin itself or molecules bound to hemozoin, such as DNA, activate TLR9 (Liehl and Mota, 2012). In fact, the AT-rich *Plasmodium* genomic DNA, a feature shared by many pathogens including *Schistosoma*, was described to be immunomodulatory *in vitro*, but this response is apparently TLR9-independent (McCutchan et al., 1984; Sharma et al., 2011).

In addition to inducing pro-inflammatory responses, recognition of parasites and *Plasmodium*-infected erythrocytes is crucial for the phagocytic uptake and thus removal of parasites from the circulation by macrophages and DCs (McGilvray et al., 2000; Stevenson and Riley, 2004; Figure 3). Interestingly, although macrophages and DCs may both contribute to early pro-inflammatory cytokine responses *via* activation of PRR-mediated signaling, a recent study suggests that macrophage responsiveness is strongly compromised upon phagocytosis of *P. falciparum*- or *P. berghei*-infected erythrocytes or free merozoites due to pronounced phagosomal acidification (Wu et al., 2015). Instead, DCs contribute to increased serum levels of pro-inflammatory cytokines early during *P. berghei* infection, including IL-6, IL-12p40, and TNF (Wu et al., 2015).

Apart from parasite-derived stimuli, host-derived DAMPs such as urate crystals, heme, and microvesicles released from damaged host cells may activate the innate immune system, although so far this has only been demonstrated for microvesicles (Gazzinelli et al., 2014). *Plasmodium falciparum*-infected erythrocyte-derived microvesicles have been reported to induce TNF and IL-10 from monocyte-derived human macrophages *in vitro*, potentially through phagocytic uptake of microvesicles (Mantel et al., 2013). In line with this observation, plasma microparticles derived from *P. berghei*-infected mice stimulated bone marrow-derived macrophages to secrete TNF *in vitro*, and TNF induction was reported to be TLR4-dependent (Couper et al., 2010). In humans, increased numbers of plasma microparticles have been detected during *P. falciparum* and *P. vivax* infection, and microparticle numbers were higher in severe malaria cases, including CM, than in uncomplicated *P. falciparum* infections (Campos et al., 2010; Nantakomol et al., 2011; Sahu et al., 2013). Furthermore, two studies point toward infection-induced alterations in the microvesicle cargo, suggesting that not only microvesicle frequency but also content are relevant in inducing pro-inflammatory responses (Couper et al., 2010; Tiberti et al., 2016). Together, these studies indicate that microvesicles might contribute to CM pathogenesis and to other manifestations of severe malaria, and the association with pro-inflammatory immune responses warrants further investigations.

Although parasite-derived stimuli have been repeatedly reported to induce pro-inflammatory responses, the real qualitative and quantitative nature of the stimuli remains inadequately understood and may point toward a complex synergistic effect of multiple stimuli. Pro-inflammatory cytokines such as TNF, IL-1α (Kwiatkowski et al., 1990; Tchinda et al., 2007), IFN-γ and IL-12p40 (Hermsen et al., 2003), as well as chemokines, including IL-8/CXCL8 (Hermsen et al., 2003), platelet factor 4 (PF4)/CXCL4, and IP-10/CXCL10 (Wilson et al., 2011), are clearly elevated during *P. falciparum* infection. Several cytokines and chemokines, including TNF and CXCL10, have been found to be associated with CM severity (Kwiatkowski et al., 1990; Wilson et al., 2011), while a more recent study reported that neither plasma nor cerebrospinal fluid (CSF) TNF concentration were indicative of CM-associated mortality, yet elevated levels of TNF in CSF of pediatric CM cases were associated with long-term neurologic and cognitive deficits (Shabani et al., 2017). Consequently, it remains to be conclusively determined which cytokines and/or chemokines present suitable prognostic signatures of disease progression.

While many early studies focused on the identification of single inflammatory cytokines critically involved in malaria pathology, it is likely that numerous immune players are modulated during the course of a *Plasmodium* infection - sequentially and/or simultaneously. Accordingly, it is conceivable that a complex interplay of immune mediators contributes to the development of severe malaria in general, and to CM pathogenesis in particular. Several studies have addressed this issue by systematically analyzing pro- and anti-inflammatory markers. Since these studies included patients from different study sites of varying age and at various time points of infection, the cytokines identified to be associated with disease severity varied substantially, and the combination of selected analytes was heterogenous between studies (Prakash et al., 2006; Jain et al., 2008; Thuma et al., 2011). An early study reported two clusters of cytokines associated with mild and cerebral malaria, respectively, in *P. falciparum*-infected adults (Prakash et al., 2006). According to this study, IFN-γ, IL-2, IL-5, IL-6 and IL-12 were increased in mild malaria whereas TGF-β, TNF, IL-10 and IL-1β were particularly elevated in CM. In a study cohort of *P. falciparum*-infected children and adults, serum TNF levels did not correlate with disease severity, and instead IP-10/CXCL10, sTNF-R2, and sFas were proposed as biomarkers of CM severity and mortality (Jain et al., 2008). Additional cytokines were elevated in malaria cases compared to healthy controls and included IL-1ra, IL-10, IL-8/CXCL8, and macrophage inflammatory protein 1β (MIP-1β)/CCL4 (Jain et al., 2008). In two cohorts of *P. falciparum*-infected children, TNF concentration was slightly, albeit non-significantly, elevated in CM compared to severe anemia cases (Thuma et al., 2011; Mandala et al., 2017). In the study by Thuma et al. (2011) conducted in Zambia, IL-10, IL-1α, IL-6, and IP-10/CXCL10 plasma levels were higher in children suffering from CM than in children with severe anemia (Thuma et al., 2011). In line with these findings, Mandala et al. (2017) found higher IL-10 and IL-6 serum levels in Malawian children suffering from CM compared to those with severe anemia, while IFN-γ and IL-8/CXCL-8 were also elevated in pediatric CM cases.

In summary, cytokine profiling continues to aid in identifying distinct patterns of pro- and anti-inflammatory cytokines and chemokines in CM patients. Although a common cytokine/chemokine signature associated with CM severity has not yet been identified, which is in part due to the fact that the combination of markers investigated varies among studies, collectively, these studies point toward important roles for certain immunoregulatory molecules in modulating CM severity. In order to describe how inflammatory mediators associated with *Plasmodium* infection may contribute to CM pathogenesis, we will highlight their roles in endothelial activation, blood-brain barrier permeability, and neuroinflammation, by drawing on findings obtained from *in vitro* studies and the murine ECM model.

## Inflammation and endothelial activation

**Figure 4:**
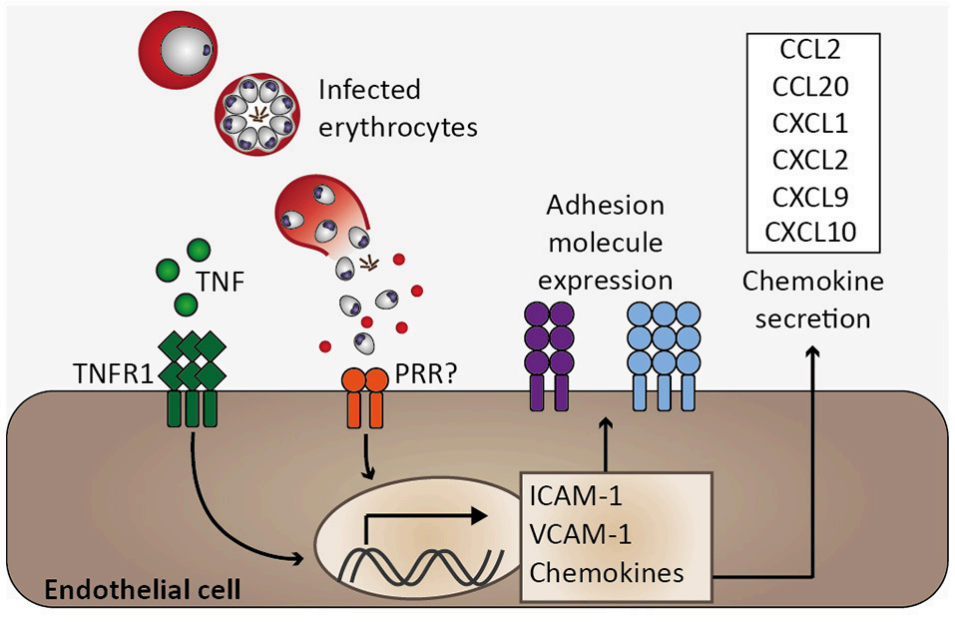
Endothelial activation and chemokine secretion. A characteristic feature of *Plasmodium* infection is endothelial activation, which is likely induced by elevated serum tumor necrosis factor (TNF) levels. Binding of TNF to its receptor (TNFR1) induces transcription of adhesion molecules, including ICAM-1 and VCAM-1, as well as chemokines (Pober and Sessa, 2007). Endothelial activation might be directly induced by infected erythrocytes, possibly through activation of pattern recognition receptors (PRR), resulting in elevated expression of ICAM-1 and chemokine secretion (Viebig et al., 2005; Tripathi et al., 2006, 2009; Chakravorty et al., 2007).

Functions of healthy endothelium include anti-coagulant properties through inhibiting platelet adhesion and aggregation, regulation of blood flow by releasing nitric oxide, controlling endothelial permeability, preventing extravasation of plasma proteins to tissue, and preventing leukocyte adhesion through suppressing adhesion molecule expression and sequestering chemokines within Weibel-Palade-bodies (Pober and Sessa, 2007). Under inflammatory conditions such as systemic inflammation during *Plasmodium* infection, endothelial activation may seriously impair endothelial function (Figure 4). A hallmark of endothelial activation is the expression of adhesion molecules such as VCAM-1 and ICAM-1 on the endothelial cell surface. Systemic endothelial activation was reported in *P. falciparum*-infected and in sepsis patients based on plasma levels of soluble adhesion molecules (Turner et al., 1998). Moreover, immunohistochemical analysis of fatal *P. falciparum*-infected CM cases revealed that expression of ICAM-1 was most pronounced in the brain microvasculature compared to other organs and biopsies from non-malaria cases (Turner et al., 1994). In good agreement, ICAM-1 staining was described to be more pronounced on brain endothelial cells from *P. berghei* (strain ANKA)-infected mice during ECM than in those isolated from *P. yoelii*-infected (non-ECM) mice (Grau et al., 1991). Accordingly, *Icam1*-deficiency protected mice from *P. berghei*-induced ECM (Favre et al., 1999). Considering that adhesion molecules are thought to promote binding of infected erythrocytes and leukocytes to endothelial cells, and that human as well as murine CM is associated with intravascular accumulation of leukocytes in the brain (Hunt and Grau, 2003), these findings signify a critical role for endothelial activation in CM pathogenesis.

Among other factors, endothelial activation may be induced by inflammatory cytokines. TNF and lymphotoxin α (LTα) activate human endothelial cells *in vitro* (Cavender et al., 1989; Pober and Cotran, 1990). Similarly, IFN-γ, IL-1α, and IL-1β function in endothelial activation (Pober and Cotran, 1990; Bauer et al., 2002). These pro-inflammatory cytokines have been found to be elevated in serum or plasma of *P. falciparum*-infected patients and TNF as well as IL-1α and IL-1β were described to be associated with CM severity (Prakash et al., 2006; Thuma et al., 2011). Consequently, endothelial activation observed in *P. falciparum* infection might in part be mediated by these cytokines. In accordance with a proposed role for endothelial activation in CM, the pathology observed in murine ECM is associated with a T helper 1 (Th 1) immune response, and cytokines, such as IFN-γ, TNF, and LTα, and immune cells, e.g., CD4^+^ and CD8^+^ T cells together with NK cells are involved in ECM (Yanez et al., 1996; Lucas et al., 1997; Engwerda et al., 2002; Hunt and Grau, 2003; Schofield and Grau, 2005; Langhorne et al., 2008). Of note, despite an association of TNF with disease severity in CM, *Tnf*-deficient mice were not protected from ECM (Engwerda et al., 2002), and blocking of TNF by anti-TNF antibodies or pentoxifylline did not improve survival in human CM (Di Perri et al., 1995; van Hensbroek et al., 1996). Consequently, blocking TNF was demonstrated to be insufficient to prevent fatal CM, and, therefore, additional mechanisms are likely to critically contribute to CM pathogenesis. Moreover, these early therapeutic interventions highlight the complexity of this malaria syndrome, indicating that single serum cytokines associated with CM severity do not necessarily translate into therapeutic approaches. Notably, elevated serum TNF in particular may represent a secondary immune response, which might further amplify severity while not being critical in initiating CM pathogenesis.

Apart from the induction of endothelial activation by pro-inflammatory cytokines, endothelial cells are an important part of the innate immune response since they recognize PAMPs through expression of PRR such as TLR and NLR. Endothelial cells were found to secrete pro-inflammatory cytokines, including IL-1α, IL-1β, or IL-6, as well as immunomodulatory cytokines, namely IL-10 and TGF-β, and chemokines, e.g., monocyte chemoattractant protein 1 (MCP-1)/CCL2, RANTES/CCL5, and IL-8/CXCL8, upon stimulation by pro-inflammatory cytokines or lipopolysaccharide (LPS) *in vitro* (Mai et al., 2013). Notably, microvascular endothelial cells derived from subcutaneous adipose tissue of patients with uncomplicated malaria and fatal CM were demonstrated to differ in their endothelial inflammatory response to TNF stimulation *ex vivo* in that MCP-1/CCL2 and IL-6 were induced to a larger extent in endothelial cells derived from CM patients. Consequently, it was proposed that inter-individual differences in the endothelial response to inflammation might account for CM severity (Wassmer et al., 2011). These results support the notion that the pro-inflammatory microvascular environment during *P. falciparum* infection might be enhanced by endothelial cells. Interestingly, human brain microvascular endothelial cells have been described to phagocytose *P. berghei* merozoites *in vitro* (Howland et al., 2015b). Furthermore, upon co-culture with *P. falciparum*-infected erythrocytes, human endothelial cell lines were reported to upregulate ICAM-1 expression (Viebig et al., 2005; Tripathi et al., 2006), to increase transcription of *CCL20, CXCL1, CXCL2, CXCL8*, and *IL6* (Tripathi et al., 2009), and to secrete MCP-1/CCL2, MIP-3α/CCL20, and IL-8/CXCL8 (Viebig et al., 2005; Chakravorty et al., 2007) (Figure 4). Together, these *in vitro* observations suggest that endothelial cells are potentially directly involved in the immune response to *Plasmodium* infection. Since leukocyte sequestration in the microvasculature of the brain was described in human CM and murine ECM (Hunt and Grau, 2003), local chemokine gradients originating from brain endothelial cells might orchestrate leukocyte migration and thus promote local inflammation, thereby contributing to the development of CM.

Although the extent of the contribution of endothelial cell-derived chemokines in the process of leukocyte accumulation in brains of patients suffering from CM and of ECM mice remains to be established, a number of clinical studies reported that several chemokines are elevated in serum and CSF of CM patients, thereby providing a potential link between serum or CSF chemokine levels and progression from mild malaria to CM. Chemokines that were elevated in human CM cases included MCP-1/CCL2, MIP-1β/CCL4, PF4/CXCL4, IL-8/CXCL8, and IP-10/CXCL10 (Jain et al., 2008; Wilson et al., 2011). Particularly, MCP-1/CCL2, Eotaxin/CCL11, and IP-10/CXLC10 were reported to be indicative of disease severity (Armah et al., 2007; Jain et al., 2008; Thuma et al., 2011; Wilson et al., 2011). Moreover, *post mortem* MIP-1β/CCL4, IL-8/CXCL8, and IP-10/CXCL10 levels were significantly elevated in cerebrospinal fluid (CSF) of fatal *P. falciparum*-induced CM cases when compared to fatal severe malarial anemia cases and non-malaria deaths (Armah et al., 2007). In another study, IL-8/CXCL8 was elevated in CSF of non-fatal CM cases of *P. falciparum*-infected children (John et al., 2008b), while MCP-1/CCL2, MIP-1α/CCL3, MIP-1β/CCL4, and RANTES/CCL5 levels were comparable to malaria-free controls. The relevance of certain chemokines in cerebral malaria pathogenesis was demonstrated in the murine ECM model, in which *Cxcl4*-, *Cxcl9*-, or *Cxcl10*-deficiency resulted in reduced ECM-associated mortality (Campanella et al., 2008; Srivastava et al., 2008; Nie et al., 2009). Notably, PF4/CXCL4 was elevated in plasma of *P. berghei* ANKA-infected mice and demonstrated to induce TNF secretion from peritoneal macrophages and T cells *in vitro*, and *Cxcl4*-deficiency resulted in reduced serum TNF and IFN-γ levels during *P. berghei* ANKA-infection (Srivastava et al., 2008), indicating that PF4/CXCL4 contributes to establishing a pro-inflammatory environment, which might amplify further immune responses and could thereby promote CM pathogenesis. Furthermore, protection from ECM in *Cxcl10*-deficient mice was associated with a decrease in leukocyte sequestration in brains of *P. berghei* ANKA-infected mice, while parasite-specific CXCR3^+^ T cells were increased in spleens of *Cxcl10*-deficient compared to WT mice (Nie et al., 2009), suggesting that IP-10/CXCL10-mediated recruitment of CXCR3^+^ T cells to the brain might contribute to the development of ECM. A similar phenomenon might account for a decrease in ECM-associated mortality described for *Cxcl9*-deficient mice (Campanella et al., 2008), since recruitment of CXCR3-expressing cells can also be initiated by monokine induced by IFN-γ (MIG)/CXCL9. However, the precise mechanism by which MIG/CXCL9 contributes to ECM pathogenesis is yet to be determined. Apart from studies using specific gene deletions, chemokine transcripts were found to be induced to a higher extent in brains of ECM- compared to non-ECM mice. These transcripts included *Ccl2, Ccl3, Ccl4, Ccl5, Cxcl1, Cxcl9*, and *Cxcl10* (Miu et al., 2008; Van den Steen et al., 2008), supporting the finding that apart from NK and T cells, monocytes and neutrophils also sequester in the microvasculature (Renia et al., 2012), e.g., through recruitment by MCP-1/CCL2 and keratinocyte chemoattractant (KC)/*Cxcl1*, respectively. Nevertheless, studies to identify the cell types producing these chemokines in the brain are very limited. For instance, CXCL9 was demonstrated to be expressed by endothelial cells, while the source(s) of CXCL10 in the brain during *P. berghei* infection remain to be conclusively determined, and could include neurons, astrocytes, or endothelial cells (Campanella et al., 2008; Miu et al., 2008) as well as recruited monocytes (Ioannidis et al., 2016).

**Figure 5:**
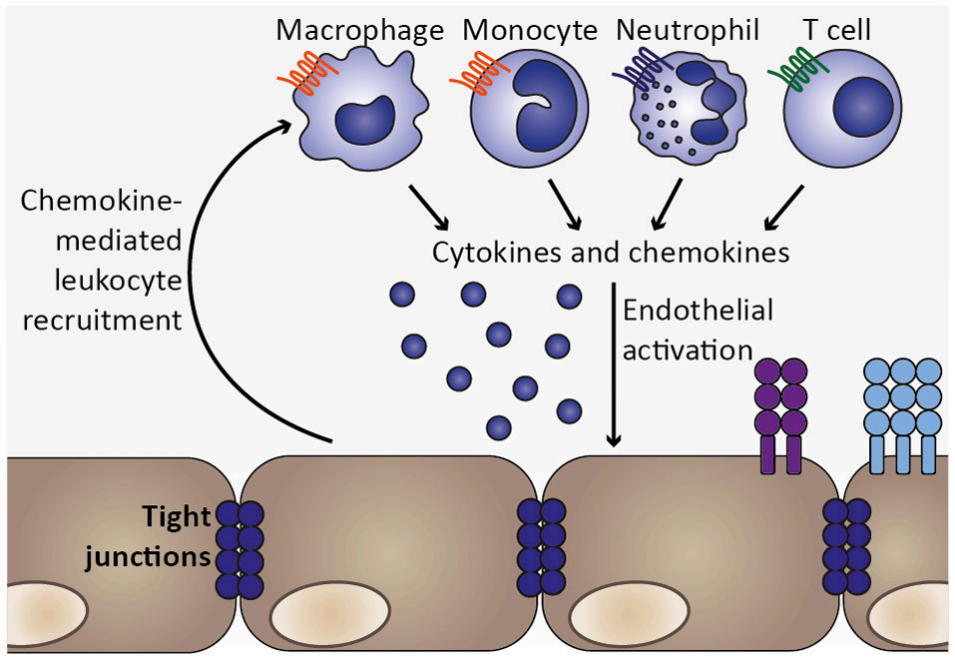
Chemokine-mediated leukocyte recruitment and progression of local inflammation. As a consequence of endothelial activation by pro-inflammatory cytokines, such as TNF, and infected erythrocytes, chemokines are secreted from endothelial cells, which initiate recruitment of leukocytes expressing the respective chemokine receptors, including macrophages, monocytes, neutrophils, and T cell. These cell types were also found to sequester in the microvasculature during human CM as well as murine ECM (Hunt and Grau, 2003; Renia et al., 2012). Upon arrival at the site of the inflammatory insult, these leukocyte subsets may in turn secrete cytokines as well as chemokines, thereby further promoting endothelial activation and leukocyte recruitment. Thus, a feed-forward loop is initiated which exacerbates local inflammation in the brain.

Together, activated endothelial cells likely contribute to local inflammation by secreting cytokines and chemokines, thereby recruiting leukocytes, including monocytes, macrophages, neutrophils and T cells, which accumulate in brains of mice and humans during ECM and CM, respectively (Renia et al., 2012; Storm and Craig, 2014). Since these cell types might secrete cytokines and chemokines themselves, local inflammation and endothelial activation could be further exacerbated (Figure 5). For instance, potential endothelial cell-induced recruitment of neutrophils and monocytes expressing IP-10/CXCL10 may promote further recruitment of CXCR3^+^ cells such as NK and T cells to the brain (Ioannidis et al., 2016).

## Endothelial activation and blood-brain barrier integrity

The blood-brain barrier is comprised of endothelial cells forming a continuous barrier through tight junctions, a basement membrane and astrocytes, which are in direct contact with neurons and microglia. This composition is critical to minimize local inflammation and neuronal damage (Obermeier et al., 2013). In the course of *Plasmodium* infection, endothelial activation may progress to vascular permeability and loss of blood-brain barrier integrity, as indicated by hemorrhages in brains of CM patients and extravasation of dyes or antibodies into the brain parenchyma in ECM (Renia et al., 2012). Although the extent of pathological events related to blood-brain barrier function in human CM is variable, dysfunction of the blood-brain barrier appears to be associated with progression of cerebral disease (Medana and Turner, 2006). Histology of brain sections from fatal human CM cases revealed a redistribution of the tight junction proteins occludin, vinculin, and zonula occludens 1 (ZO-1), which are central to blood-brain barrier integrity (Brown et al., 1999). Moreover, immunohistochemistry of brain sections derived from pediatric fatal CM cases indicated blood-brain barrier impairment in areas containing sequestered *P. falciparum*-infected erythrocytes, where they were associated with focal loss of endothelial intercellular junctions (Brown et al., 2001). Additionally, *in vitro* studies have demonstrated a decrease in endothelial resistance upon addition of *P. falciparum*-infected erythrocytes to endothelial cells (Tripathi et al., 2007; Jambou et al., 2010). This model, however, only partially reflects the response at the blood-brain barrier due to lack of barrier components such as astrocytes and pericytes (Medana and Turner, 2007).

**Figure 6:**
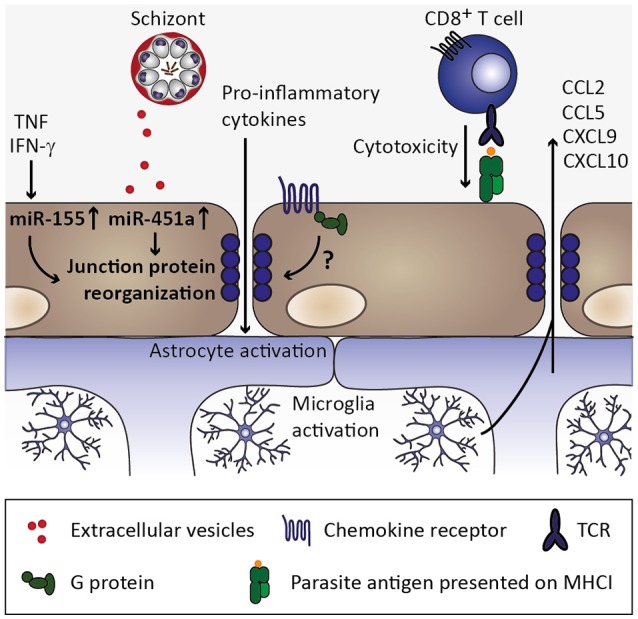
Endothelial permeability and neuroinflammation. Through continued inflammatory insults toward endothelial cells by, for instance, circulating tumor necrosis factor (TNF) and interferon γ (IFN-γ), miR-155 might be upregulated in endothelial cells. Along with uptake of miR-451a from *P. falciparum*-infected erythrocyte-derived extracellular vesicles, reorganization of tight junction proteins such as zonula occludens 1 (ZO-1) is induced and could contribute to endothelial permeability during cerebral malaria (Lopez-Ramirez et al., 2014; Mantel et al., 2016). Additionally, chemokine receptors might induce redistribution of tight junction proteins in a G protein-dependent manner (Stamatovic et al., 2003; Song and Pachter, 2004; Yao and Tsirka, 2011), while the contribution of chemokine-induced opening of tight junctions is less clear in the context of cerebral malaria. CD8^+^ T cell-mediated cytotoxity toward endothelial cells through recognition of parasite antigen presented on MHC class I molecules on endothelial cells likely contributes substantially to blood-brain barrier permeability during cerebral malaria (Howland et al., 2015a,b). Consequently, pro-inflammatory cytokines enter the brain parenchyma and could thereby activate astrocytes and microglia, which in turn could secrete chemokines (Capuccini et al., 2016) and thus promote leukocyte recruitment and local inflammation.

Interestingly, MCP-1/CCL2 induces redistribution of tight junction proteins and increases endothelial permeability *in vitro* (Stamatovic et al., 2003; Song and Pachter, 2004; Yao and Tsirka, 2011). Thus, chemokines may contribute to organ-specific inflammation by inducing signals that promote endothelial permeability (Figure 6). The roles of the respective chemokine receptors are less clear in this context. This is exemplified in the MCP-1/CCL2 receptor CCR2. *Ccr2*-deficiency abrogated CCL2-induced endothelial permeability *in vitro* (Stamatovic et al., 2003), but did not protect against ECM (Belnoue et al., 2003), indicating that other chemokines and/or additional mechanisms induce blood-brain barrier permeability.

Along with the observed redistribution of endothelial tight junction proteins and loss of intercellular junctions, growing evidence from the murine ECM model suggests that CD8^+^ T cells are primary mediators in CM disease pathogenesis by contributing considerably to the loss of blood-brain barrier integrity (Howland et al., 2015a). Indeed, the accumulation of CD8^+^ T cells in brains of *P. berghei* ANKA-infected mice appears to be critical for the development of ECM (Villegas-Mendez et al., 2012), and blood-brain barrier disruption was proposed to be a consequence of CD8^+^ T cell-mediated cytotoxicity toward endothelial cells cross-presenting parasite antigen (Howland et al., 2015b) (Figure 6). Recruitment of CD8^+^ T cells to the brain of infected mice has been described to be partly mediated by expression of the chemokine receptor CXCR3 and its IFN-γ-inducible ligands MIG/CXCL9 as well as IP-10/CXCL10 (Hansen et al., 2007; Villegas-Mendez et al., 2012). Indeed, NK cell-derived IFN-γ has been demonstrated to be crucial for the induction of CXCR3 expression on T cells and subsequent T-cell migration to the brain of *P. berghei* ANKA-infected mice (Hansen et al., 2007). In line with these findings, brain transcripts of *Cxcr3* were reduced in mice deficient for IFN-γR1 in comparison to wild type mice infected with *P. berghei* ANKA. Additionally, while expression of MIG/CXCL9 and IP-10/CXCL10 is induced in brains of *P. berghei* ANKA-infected wild type mice, expression levels of these chemokines in *Ifngr1*-deficient mice were similar to those of uninfected mice (Palomo et al., 2013). Collectively, these findings suggest that IFN-γ-induced signaling is crucial for chemokine-mediated leukocyte recruitment to the brain during ECM, while it remains to be established to which extent activated endothelium might contribute to this chemokine response.

Importantly, *Cxcr3*-deficient mice were less likely to succumb to ECM (Campanella et al., 2008; Miu et al., 2008), and numbers of CD4^+^ and CD8^+^ T cells in brains of *Cxcr3*-deficient mice were reported to be reduced compared to wild type mice during *P. berghei* ANKA infection. However, the extent of CD8^+^ T cell recruitment was similar in *Cxcr3*-deficient mice which were either resistant or susceptible to ECM (Miu et al., 2008). These results indicate that the quantity of CD8^+^ T cells in brains of *P. berghei* ANKA-infected mice is not critical for the development of ECM. Rather, additional aspects such as CD8^+^ T-cell specificity toward parasite antigen presented on endothelium, expression levels of key cytotoxic effector molecules perforin and granzyme B by CD8^+^ T cells, and localization of these CD8^+^ T cells within the brain might play a substantial role in the development of ECM (Miu et al., 2008; Haque et al., 2011; Howland et al., 2015b; Huggins et al., 2017). Nevertheless, it remains to be determined why susceptibility to ECM among *Cxcr3*-deficient mice is variable. Moreover, these findings indicate that additional mechanisms other than CD8^+^ T cell-mediated cytotoxicity might be involved in the loss of blood-brain barrier integrity.

In fact, in addition to CD8^+^ T cells and chemokines, extracellular vesicles derived from *P. falciparum*-infected erythrocytes were recently implicated in blood-brain barrier permeability *in vitro* (Mantel et al., 2016; Figure 6). These extracellular vesicles contained miRNA miR-451a, which, upon endocytosis by endothelial cells, correlated with reduced endothelial caveolin-1 expression. Interestingly, MCP-1/CCL2-induced redistribution of tight junction proteins and concomitant endothelial permeability (Stamatovic et al., 2003) was also accompanied by a decrease in caveolin-1 protein (Song and Pachter, 2004), thus pointing toward a common underlying mechanism in the induction of endothelial permeability by CCL2 and extracellular vesicles derived from *P. falciparum*-infected erythrocytes. Since sequestration of infected erythrocytes in the microvasculature is likely promoted by expression of adhesion molecules upon endothelial activation, locally increased release of *P. falciparum*-infected erythrocyte-derived extracellular vesicles carrying miRNA miR-451a might be an additional mechanism involved in blood-brain barrier permeability in CM.

In addition to miR-451a, another miRNA, miR-155, was implicated in inflammation-associated blood-brain barrier permeability. Upon stimulation of human brain endothelial cells with pro-inflammatory cytokines TNF and IFN-γ *in vitro*, miR-155 was found to be upregulated. The tight junction protein claudin-1 was reported to be among the candidate targets of miR-155, and cytokine stimulation or miR-155 overexpression resulted in reorganization of the tight junction protein ZO-1 along with increased endothelial permeability (Lopez-Ramirez et al., 2014), indicating that cytokines such as TNF and IFN-γ might directly contribute to blood-brain barrier permeability through induction of regulatory miRNAs. Interestingly, levels of miR-155 were recently reported to be elevated in extracellular vesicles in the circulation of *P. berghei* ANKA-infected mice, and *miR-155*-deficiency resulted in preservation of blood-brain barrier integrity and reduced ECM-associated mortality (Barker et al., 2017). Notably, plasma concentrations of IL-6, IFN-γ, and MCP-1/CCL2 were significantly elevated in *P. berghei* ANKA-infected *miR-155*-deficient compared to wild type mice in this study, suggesting that miR-155 might have additional targets other than tight junction proteins. Importantly, whether miR-155-carrying extracellular vesicles are derived from activated endothelium, and their impact on tight junction reorganization in the context of ECM, remain to be determined. Nevertheless, inhibition of the function of miR-155 might present a useful target for therapeutic approaches (Barker et al., 2017). These processes of chemokine induction, microvascular sequestration of infected erythrocytes and leukocytes, and release of extracellular vesicles carrying regulatory miRNAs are most likely not exclusive to the brain. However, endothelial permeability is likely more detrimental in the brain than in other organs and, hence, cerebral malaria may be the most severe manifestation of these processes. Additionally, brain endothelial cells have been described to express comparably low levels of thrombin-binding thrombomodulin, and excess of unbound thrombin may further promote local endothelial activation by inducing further expression of adhesion molecules (Clark et al., 2006).

Together, cytokine- or *Plasmodium*-induced endothelial activation may lead to chemokine induction and leukocyte recruitment as well as sequestration of infected erythrocytes, which may act synergistically in promoting endothelial permeability. Nevertheless, the precise molecular mechanisms that trigger loss of blood-brain barrier integrity in CM are incompletely understood and need to be further investigated.

## Neuroinflammation

Upon disruption of the blood-brain barrier, cytokines, chemokines, and soluble parasite products might enter the brain parenchyma and, thereby, activate astrocytes and microglia, and result in symptoms of neuroinflammation in the absence of extravasation of infected erythrocytes or leukocytes into the brain parenchyma (Combes et al., 2010). Indeed, activation of microglia and astrocytes has been observed in murine and human CM (Hunt et al., 2006; Combes et al., 2010). For instance, transcriptome analysis of microglia isolated from *P. berghei*-infected mice revealed that several chemokines as well as transcripts related to type I IFN signaling were differentially upregulated (Capuccini et al., 2016). This finding was confirmed *in vitro* by stimulation of a murine microglia cell line with IFN-β, which resulted in secretion of MCP-1/CCL2, RANTES/CCL5, MIG/CXCL9, and IP-10/CXCL10. Moreover, stimulation of human primary astrocytes with a combination of IFN-γ and LTα synergistically induced IP-10/CXCL10 secretion *in vitro* (Bakmiwewa et al., 2016). Additionally, co-culture of *P. berghei*-infected erythrocytes with a mixed astrocyte-microglia culture resulted in phagocytic uptake of infected erythrocytes and of parasitized erythrocyte-derived microvesicles by microglia and astrocytes, respectively, which in turn was associated with an induction in IP-10/CXCL10 secretion (Shrivastava et al., 2017). Furthermore, astrocytes and microglia may secrete various cytokines and chemokines upon activation (Dong and Benveniste, 2001; Medana et al., 2001). Consequently, loss of blood-brain barrier integrity and subsequent activation of microglia and astrocytes might result in further chemokine-mediated recruitment of leukocytes to the brain and subsequent amplification of inflammation (Figure 6). Additionally, activation of microglia might induce expression of FasL, which, upon binding to Fas expressed on astrocytes, could induce astrocyte damage (Hunt et al., 2006). However, to our knowledge, this has so far not been demonstrated in the context of CM. Since astrocytes are critically involved in maintaining blood-brain barrier properties and survival of neurons (Combes et al., 2010), their functional impairment might disrupt neuronal activity and could thereby account for the neurological impairment observed in some CM cases (Hunt et al., 2006).

## Summary and perspectives

The human and murine immune system are in part strikingly different (Stevenson and Riley, 2004). For instance, IL-8/CXCL8 was reported to be associated with cerebral malaria (Armah et al., 2007; John et al., 2008b), while this chemokine is not expressed in mice (Viola and Luster, 2008). However, KC/*Cxcl1* is considered a functional homolog of IL-8/CXCL8 in mice (Hol et al., 2010), which mediates neutrophil recruitment, and *Cxcl1* transcripts were reported to be elevated in brains of ECM compared to non-ECM mice (Miu et al., 2008), suggesting that KC/*Cxcl1* might be similarly involved in ECM pathogenesis. Yet, the precise contribution of IL-8/CXCL8 and KC/*Cxcl1* to human CM and murine ECM, respectively, needs to be further investigated. Moreover, the murine ECM model shares several features with human CM, including aspects of histopathology and inflammatory responses. Importantly, mechanistic insights can only be gained from the murine ECM model, and many observations are in remarkably good agreement with clinical data obtained from *P. falciparum*-induced CM. Although a comprehensive representation of the events leading to CM pathogenesis remains elusive, a working model of an inflammatory cascade leading to CM is conceivable (Figure 1).

Upon establishing liver stage infection, a first type I IFN response is mounted by hepatocytes, leading to a primary activation of IFN-γ-producing NK cells (Liehl et al., 2014; Miller et al., 2014; Figure 2) and, consequently, induction of MIG/CXCL9 and IP-10/CXCL10 secretion, potentially from endothelial cells (Campanella et al., 2008; Miu et al., 2008). Upon progression of the *Plasmodium* infection to the blood stages, infected erythrocytes are recognized by DCs and induce the secretion of IL-12 and TNF (Wu et al., 2015; Figure 3). IL-12 contributes to further activation of NK and differentiation of Th1 cells (Stevenson and Riley, 2004), while TNF and IFN-γ activate chemokine transcription and adhesion molecule expression on endothelial cells (Pober and Sessa, 2007; Miu et al., 2008; Griffith et al., 2014). As the infection progresses further, *Plasmodium*-infected erythrocytes are recognized by endothelial cells and induce expression of chemokines, such as MCP-1/CCL2 and IL-8/CXCL8 (Viebig et al., 2005; Chakravorty et al., 2007; Tripathi et al., 2009; Figure 4). As a result, leukocytes are recruited, including monocytes, macrophages, neutrophils, as well as T cells, and initiate a local inflammatory response (Renia et al., 2012; Storm and Craig, 2014; Figure 5). These cell types secrete chemokines, thereby amplifying the response leading to further leukocyte recruitment and intensifying local inflammation. Additionally, endothelial cells phagocytose merozoites and parasite material released during schizont rupture and present parasite antigens to CD8^+^ T cells (Howland et al., 2015b; Figure 6), which may result in targeted elimination of antigen-presenting endothelial cells and, thus, cause damages to the endothelial lining of the blood-brain barrier. This process can be further exacerbated by openings of tight junctions mediated by chemokines and extracellular vesicle-derived miRNAs (Song and Pachter, 2004; Mantel et al., 2016). As a result, small molecules can enter the brain parenchyma and potentially activate brain-resident microglia and astrocytes, further amplifying local inflammation through cytokine secretion and leukocyte recruitment and impairing neuronal functionality (Hunt et al., 2006; Combes et al., 2010).

Even though the febrile response elicited during *Plasmodium* blood stage infection together with the concomitant inflammatory cytokine responses limit parasite growth and mediate the resolution of infection, imbalances in pro-inflammatory and anti-inflammatory cytokines cause progression of malaria disease to manifestations of severe malaria, such as CM, and death. Adjunctive therapies that prevent adverse effects of the immune response to *Plasmodium* infection are therefore urgently needed. Although immunomodulation is a promising approach to alleviate immune-mediated pathology, such therapies need to be designed carefully in order to maintain efficient control of parasite growth. Notably, adjunct therapies modulating chemokine responses may have fewer side-effects compared to therapies based on neutralizing cytokines (Ioannidis et al., 2014). Indeed, antibody-mediated targeting of IP-10/CXCL10 was demonstrated to result in reduced ECM-induced mortality and parasite burden in mice, which was likely mediated by retention and expansion of parasite-specific T cells in the spleen (Nie et al., 2009). Such treatments may be relevant in other contexts as well: murine *Toxoplasma* encephalitis has been described to be associated with constant expression of *Ccl2, Ccl3, Ccl4, Ccl5*, and *Cxcl10* in brains of infected mice concomitant with continuous recruitment of CD4^+^ and CD8^+^ T cells, which was not the case for mice in which the infection developed into chronic latency (Strack et al., 2002). Furthermore, targeted neutralization of single chemokines, including MCP-1/CCL2, MIP-1α/CCL3, RANTES/CCL5, or IP-10/CXCL10, resulted in protection of mice from experimental autoimmune encephalomyelitis, a murine model for multiple sclerosis (Karin and Wildbaum, 2015). These findings from other neuroinflammatory diseases highlight that chemokines might present a valuable target for intervention strategies in several diseases. However, efforts to design chemokine-based therapies are challenged by the complexity of the chemokine system as well as properties such as redundancy, pleiotropy, and speciation (Viola and Luster, 2008). In fact, most cell populations express several different chemokine receptors and thus single chemokine or chemokine receptor blockade may not affect disease outcome in certain pathological conditions.

Together, reliable biomarkers which predict disease outcome and allow for potential prophylactic measures are yet to be identified. Several clinical studies have reported potential diagnostic and prognostic biomarkers for CM, which apart from chemokines such as IP-10/CXCL10 and PF4/CXCL4 also include *P. falciparum* histidine-rich protein 2 (*Pf* HRP2), a protein which correlates with parasite biomass, as well as regulators of endothelial activation angiopoietin-1 and -2 (summarized in Sahu et al., 2015). Still, comprehensive studies with defined clinical parameters and systematic assessment of plasma levels of multiple inflammatory mediators need to be performed to determine whether distinct clusters of markers can be associated with disease severity in order to identify patients at risk of developing CM early during infection. Such studies will inform future investigations into mechanisms underlying disease pathogenesis in order to develop novel evidence-based malaria intervention strategies.

## Author contributions

All authors listed have made a substantial, direct and intellectual contribution to the work, and approved it for publication.

### Conflict of interest statement

The authors declare that the research was conducted in the absence of any commercial or financial relationships that could be construed as a potential conflict of interest.

## Abbreviations

CD: cluster of differentiation
CM: cerebral malaria
CSF: cerebrospinal fluid
DAMP: danger-associated molecular patterns
DC: dendritic cell
ECM: experimental cerebral malaria
EPCR: endothelial protein C receptor
GPI: glycosylphosphatidylinositol
ICAM: intercellular adhesion molecule
IFN: interferon
IL: interleukin
IP-10: IFN-γ-inducible protein 10
KC: keratinocyte chemoattractant
LPS: lipopolysaccharide
LT: lymphotoxin
MCP-1: monocyte chemoattractant protein 1
MIG: monokine induced by IFN-γ
MIP: macrophage inflammatory protein
NK: natural killer
NLR: NOD-like receptors
PAMP: pathogen-associated molecular pattern
PF4: platelet factor 4
*Pf* EMP1: *Plasmodium falciparum* erythrocyte membrane protein 1
PRR: pattern recognition receptor
TLR: Toll-like receptor
TNF: tumor necrosis factor
VCAM: vascular cell adhesion molecule
ZO-1: zonula occludens 1.
